# Breaking the cycle of reoccurring low back pain with integrated motivational interviewing and cognitive behavioural therapy to facilitate education and exercise advice: a superiority randomised controlled trial study protocol

**DOI:** 10.1186/s12889-024-19930-8

**Published:** 2024-09-05

**Authors:** Estelle D. Watson, Paul W. Marshall, Natalie M. V. Morrison, Niamh Moloney, Paul O’Halloran, Martin Rabey, Imran Khan Niazi, Kirk Stevens, Michael Kingsley

**Affiliations:** 1 Department of Exercise Science, Faculty of Science, University of Auckland, Auckland, New Zealand; 2 Translational Health Research Institute Western Sydney University, Sydney, Australia; 3 Faculty of Health Sciences, Curtin University, Perth, Australia; 4 School of Psychology and Public Health, La Trobe University, Melbourne, Australia; 5 Centre for Chiropractic Research, New Zealand College of Chiropractic, Auckland, New Zealand; 6 Holsworth Research Initiative, La Trobe University, Melbourne, Australia

**Keywords:** Motivational interviewing; Cognitive behavioural therapy; Low back pain

## Abstract

**Background:**

Non-specific low back pain is a common and costly global issue. Many people with low back pain live for years with ongoing symptom recurrence and disability, making it crucial to find effective prevention strategies. Motivational interviewing (MI) is an evidence-based patient-centred counselling style that helps motivate individuals to change their behaviours. In combination, MI and cognitive-behavioural therapy (MI-CBT) has the potential to yield long term improvements in pain and disability and reduce incidence of recurrence.

**Method:**

This is a two-arm superiority randomised controlled trial comparing MI-CBT and Education (*n* = 83) with Education only (*n* = 83). Participants that have recovered from a recent episode of non-specific low back pain (7th consecutive day with pain ≤ 2 on a 0–10 numeric pain rating scale) will be eligible for inclusion into the study. Both groups will receive five 30-min sessions over a 10-week period as well as the Navigating Pain booklet, homework book and a standardised exercise programme. In the intervention group, MI-CBT techniques will be used to provide individualised support, identify beliefs, and increase engagement with the resources provided. Outcomes measures include pain (current and in the last 7 days) as rated on the numeric pain rating scale. This will be used to determine recurrence (number of participants who report back pain ≥ 3 out of 10 on the numeric pain rating scale). Furthermore, self-reported (1) pain intensity; (2) pain catastrophizing; (3) fear-avoidance beliefs; (4) pain self-efficacy; (5) depression and anxiety; (6) disability will be measured. All outcomes will be measured at baseline, and again at 3-, 6-, and 12-months post allocation.

**Discussion:**

The effective delivery of self-management strategies to prevent recurrence of low back pain is an important aspect that requires urgent attention. This study will provide new information on the effectiveness of using an MI-CBT approach to facilitate self-management through education and exercise to improve low back pain outcomes. Evidence emerging from this trial has the potential to inform clinical practice and healthcare management of non-specific low back pain.

**Trial registration:**

Prospectively registered with Australian New Zealand Clinical Trials Registry: ACTRN12623000746639 (10/07/2023).

**Supplementary Information:**

The online version contains supplementary material available at 10.1186/s12889-024-19930-8.

## Introduction

Low back pain is the leading cause of disability worldwide [1–3] and is second only to the common cold as the reason for seeing a general practitioner [4]. In 2020, this condition affected 619 million people globally, with cases projected to exceed 800 million by 2050 [3]. While a significant percentage of acute back pain episodes resolve within 2 to 3 months of onset [5, 6], recurrence rates are alarmingly high, ranging from 25 to 60% at one year [7–9] leading to 20% to 30% of individuals developing chronic pain [10]. This high recurrence rate, and chronic pain trajectory, underscores the urgent need for effective prevention strategies. To address this global health challenge, clinical practice guidelines have been developed worldwide, all advocating for a biopsychosocial approach [11–13]. These guidelines emphasize initial education, advice, self-management, and the potential use of physical and psychological therapies. Patient education and exercise have shown moderate-quality evidence in alleviating acute back pain symptoms and reducing the likelihood of recurrence [11, 14]. However, achieving and maintaining adherence to these self-management strategies is often an issue [15], despite being a key component of the effectiveness of these types of treatments [16].

Given the persistently high recurrence rates, there is a compelling need to explore innovative approaches to enhance the uptake and adherence to patient education and exercise advice. The integration of Motivational Interviewing (MI) and Cognitive-Behavioural Techniques (CBT) into pain education and exercise advice emerges as a promising avenue for addressing the burden of low back pain recurrence.

Patient education typically focuses on improving self-management by providing information about the nature of pain and encouraging the continuation of normal activities [12]. Worldwide, a range of pain education booklets for patients have been developed by health professionals, researchers, and professional organisations (e.g., Navigating Pain by the New Zealand Pain Society). Recommended exercise prescription varies, encompassing different types of exercise (e.g., advice to stay active, stretching, aerobic exercise, motor control exercises, yoga, tai-chi) and modes of delivery (group, individual, self-guided, supervised) [13]. Some evidence suggests that education and exercise can reduce the recurrence of back pain by up to 45% at 12-months [17]. However, the efficacy of education and exercise for patients with acute low back pain is equivocal [8, 9]. For example, individualised pain education was no different from placebo (listening, showing interest, giving attention) for both acute and 12-month reductions in pain as well as healthcare seeking behaviour [8]. Similarly, 12-weeks of individualised exercise and education which covered anatomy, function, back pain risk factors, and activity, did not differ significantly from providing a ‘Managing Back Pain’ booklet in preventing back pain recurrence at 12-months [9]. The lack of positive outcomes in these studies could be attributed to several factors, including suboptimal exercise adherence, and not addressing individual psychosocial factors or pain beliefs. Neither study supplemented their patient interactions and delivery of education or advice with a behavioural technique such as MI, which provides for a bespoke delivery of information [18].

MI is a directive, evidence-based patient-centred counselling style that supports an individual’s motivation to change [19, 20]. MI has demonstrated effectiveness in promoting health-related behaviour change across various populations and health conditions [18, 21–26]. In people with acute and sub-acute low back pain, MI has been shown to improve functional capacity when used as an adjunct to physiotherapy [27]. Although some evidence has explored the use of MI for individuals with chronic low back pain [28–30], no study has investigated the potential of MI to augment patient education and exercise for preventing back pain recurrence. MI might play a pivotal role in preventing back pain recurrence and improving fidelity of evidence-based practices [20].

Cognitive behavioural therapy (CBT) is a psychosocial therapeutic method of behavioural change focussed on restructuring maladaptive beliefs and emotions as well as improving coping skills and behaviours [31]. It has been shown to be effective in improving pain, physical function, quality of life and pain catastrophizing beliefs [31–33], and is a recommended first line of treatment for chronic low back pain [11]. Additionally, combining MI with complementary treatments such as CBT, that is MI-CBT, has been shown to improve health-related outcomes such as physical activity and body composition [24]. However, to our knowledge, no previous studies have examined the potential of MI-CBT to facilitate established self-management strategies (education and exercise) for reducing back pain recurrence after an acute episode of low back pain.

This protocol paper outlines the design of a randomized controlled trial, which aims to assess the effectiveness of patient education and exercise advice facilitated by MI and CBT techniques (*Edu* + *MI-CBT*) compared to education and exercise (*Edu only*) in preventing low back pain recurrence among individuals who have recently recovered from an acute episode. The primary objectives are to examine the proportion of patients reporting pain ≥ 3 out of 10 at 3, 6 and 12 months [9] and the pain intensity reported at 12 months, between the two groups. Secondary objectives include examining changes in pain catastrophizing, fear-avoidance beliefs, pain self-efficacy, depression and anxiety, and disability, as well as monitoring ongoing treatment, medication usage and physical activity reporting. Positive findings from this trial will inform the scalable implementation of such strategies within the primary care system of New Zealand.

## Methods

**Fig. 1:**
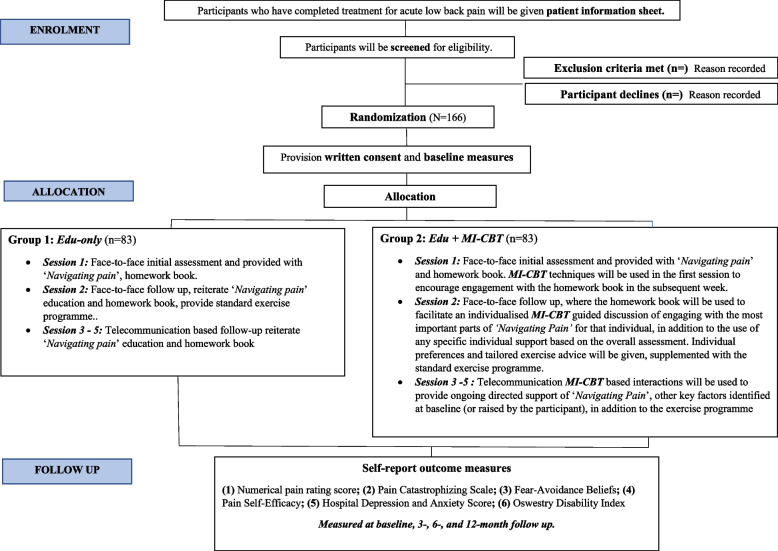
Participant flow chart

This study is a two-arm superiority randomized controlled trial comparing *Edu* + *MI/CBT* vs *Edu only* for participants who have ceased treatment from an episode of acute low back pain. Potential participants who have completed treatment for an acute episode of low back pain New Zealand Chiropractic College clinic, will be screened for eligibility. Those who meet the inclusion criteria will be randomly assigned to either *Edu* + *MI/CBT* or *Edu only*. Allocation will be concealed until after the baseline measures are collected at the first session (Fig. 1). Both groups will receive standard self-management resources, which include the “Navigating Pain” booklet, homework book and an exercise programme, and have five 30-min sessions over a 10-week period. In the intervention group, MI-CBT techniques will be used to provide individualised support, identify beliefs, and increase engagement with the resources provided. In the Education only group, participants will be provided with the resources as per standard protocols for delivering health education, where participants are directed in terms of what to do, without providing any specific MI-CBT based support. All outcome measures will be assessed at 3-, 6- and 12 months follow up. The protocol follows the ‘Standard Protocol Items: Recommendations for Interventional Trials’ (SPIRIT) guidelines (Supplementary File 1) [34].

### Participant eligibility and recruitment

Participants will be eligible to participate in the study if they are 18 years and older and have recovered (defined as ≥ 7 consecutive days with pain ≤ 2 on a 0–10 numeric pain rating scale) from a recent (i.e., pain onset within last 3-months) episode of acute non-specific low back pain (defined as pain in the area between the 12th rib and buttock crease not attributed to a specific diagnosis) and they have received treatment for this at the New Zealand College of Chiropractic Clinic.

Participants will be excluded from the study if they have any specific low back pain diagnosis (e.g., discitis, malignancy, axial spondyloarthropathy); radiating or referred pain past the buttock; previous spinal surgery; have had any back pain in the last year prior to the most recent episode that has required them to seek treatment from an allied health professional or take pain relieving medication; diagnosed with any co-morbidity that prevents participation in exercise; inadequate English language that prevents completion of outcome measure assessment or engagement in the intervention.

The study will be conducted at the community clinic of the New Zealand (NZ) Chiropractic College in Auckland, New Zealand. Participants are invited to participate in the study by Chiropractors and administrative staff at the community clinic, after completion of their low back pain treatment. Potential participants will be screened telephonically for eligibility prior to inclusion. The researchers will discuss the trial protocol and provide the participant information sheet to eligible participants (Supplementary File 2). Consent will be taken by a researcher/s trained in Good Clinical Practice (GCP). Participants will be given the opportunity to provide consent in-person, electronically, or by mail (Supplementary File 2).

### Blinding and randomisation

After consent is provided, included participants will be randomly allocated to either *Edu* + *MI-CBT* or *Edu only* using a computer-generated alphanumeric code. A researcher (IKN) not involved in the recruitment, enrolment, or data collection will generate the allocation sequence and perform the randomisation. Randomisation will use a 1:1 allocation ratio. A sealed envelope with the participant’s group assignment will be presented to the researcher at the time of the first session.

Participants will be informed that the trial is comparing two interventions, both involving provision of education and exercise, but will be blinded to method of delivery. Unblinded researchers will carry out both interventions and enter the data into coded files to ensure group membership is blinded during analysis. Researchers performing the data analysis will be blinded to the groups. There is no foreseeable requirement for unblinding participants during the study.

## Outcome assessments

All outcomes will be measured at baseline, 3-, 6- and 12-months post commencement (Table 1). Participant characteristics obtained at baseline will include age, gender, work status, ethnicity, time off work due to back pain in the last month, self-reported back pain intensity at the start of recent treatment, back pain medication use in the last month, physical activity levels in the last week, and back pain treatments undertaken in the last month.

**Table 1:**
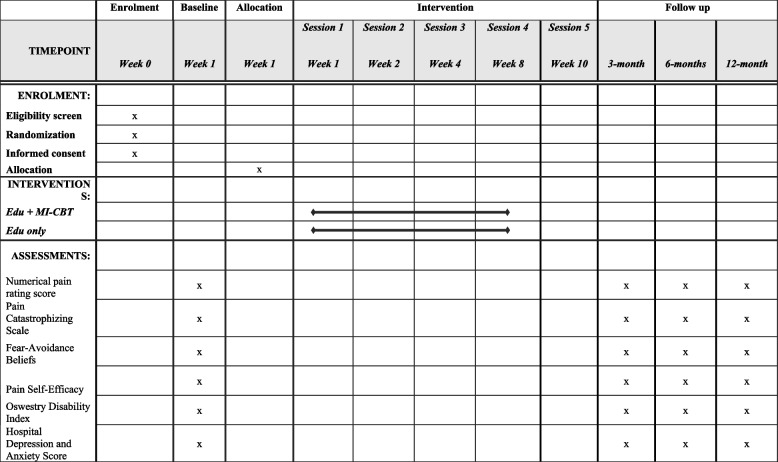
Schedule of procedures

### Primary outcomes

The primary outcome measures are proportion of participants reporting low back pain (current and in the last 7 days) ≥ 3 out of 10 (using the numeric pain rating scale, 0 (no pain) to 10 (worst possible pain) at 3, 6 and 12 months; and pain intensity (current and in last 7 days) reported at 12 months [35].

### Secondary outcomes

#### Catastrophizing

The Pain Catastrophizing Scale is a 13-item self-report questionnaire widely used to assess pain catastrophizing, with good levels of validity, reliability, internal consistency, and precision [36].

#### Fear avoidance

The Fear-Avoidance Beliefs Questionnaire (FABQ) consists of two subscales related to work activity and physical activity, where each item is scored on an ordinal 7-point Likert-type scale [37].

#### Pain self-efficacy

The Pain Self-Efficacy Questionnaire (PSEQ) is a 10-item questionnaire that allows participants to rate their confidence from 0 (not at all confident) to 6 (completely confident) in performing activities while in pain [38].

#### Mental health

The Hospital Depression and Anxiety Score (HADS) is a commonly used self-rating scale developed to assess psychological wellbeing, particularly in the domains of anxiety and depression [39].

#### Disability

The Oswestry Disability Index (ODI) is one of the most used questionnaires for examining the impact of low back pain. It assesses perceived disability in 10 activities of daily living, with each item consisting of 6 statements which are scored from 0 (least disability) to 5 (greatest disability). It has been shown to be a valid, reliable, and responsive tool that is easy to administer in the clinical setting [40].

#### Other

Other outcomes such as treatment received, medication use for low back pain, low back pain-related work absence and physical activity levels will be collected via single-item forced choice and open answer items.

## Intervention

Both groups will have two 30-min face-to-face sessions (Session 1 and Session 2) separated by approximately 1 week. In Session 1, all participants will be provided with a ‘Navigating Pain’ booklet [41] (NZ Pain Society). ‘Navigating Pain’ is a freely available resource specifically designed by health care professionals to help people in New Zealand understand and manage their pain. Content includes sections covering Understanding Pain (i.e., knowledge and definitions), Understanding Their Personal Situation (i.e., intrapersonal factors), Healthy Lifestyle Choices (i.e., physical activity and sleep), and Living Your Life (i.e., identifying support, use of pacing). Additionally, a ‘homework book’ will be provided to all participants (Supplementary File 3). This book covers five scenarios based on the sections of ‘Navigating Pain’. These include: (1) Understanding Pain, (2) Sleep, (3) Exercise, (4) Back Pain and You, and (5) Giving Advice to Future You. They are designed to explore thoughts, encourage self-reflection, and plan strategies for future pain in accordance with principles of CBT for pain presentations. In Session 2, all participants will be provided with an exercise programme for back pain. Thereafter, a further three sessions will be provided (either face-to-face or via telephone) in between weeks 4–10, and these will be used to follow up on the exercise and reiterate key messages from the education booklet as well as discuss relapse prevention strategies.

The *Edu* + *MI-CBT* intervention will begin with a face-to-face session (Session 1) which will allow participants to share their pre-back pain activities, motivations for trial participation, and prior experiences with back pain and recovery. MI techniques will be employed to develop rapport, explore motivations and barriers to self-management of pain, as well as understanding participants' unique perspectives. The homework book will also be introduced to participants in Session 1 emphasizing its role in comprehending pain beliefs, sleep habits, physical activity, and action planning (reinforcing CBT strategies). Participants' ambivalence and concerns regarding use of the homework book will be addressed collaboratively, eliciting the client’s perceived benefits of working with the book in addition to engaging in flexible goal setting for outcomes from trial participation in line with MI protocols. Session 2, in addition to the exercise program, participants will review the homework to assist in the understanding pain and physical activity scenarios. MI-CBT techniques such as open-ended questions, affirmations, reflections, and summarising, will be used to explore pain beliefs around the core message that pain does not always equate to damage. The multifaceted nature of pain perception will be explored, acknowledging that various factors, including life stressors, past experiences, and expectations, can influence pain perception. In line with an MI approach, participants' personal insights and beliefs about pain will be elicited, and discussions revolve around how this understanding impacts their own pain perception and management. Participants' thoughts on the importance of physical activity for preventing future back pain are explored, and personalized exercise advice will be offered based on their preferences and history. This approach will emphasize individualized exercise recommendations, highlighting the effectiveness of various exercise types while allowing participants to lead the discussion towards the exercise solution that suits them best. The MI-CBT follow-up sessions will offer ongoing guidance on 'Navigating Pain,' individualized support, and reinforcement of engagement in physical activity. In session 3, participants' perceptions of sleep quality will be explored. This includes addressing how participants might feel in the morning after waking, reasons for sleep changes, capacity to sleep confidence levels, and strategies for improving sleep quality (e.g., sleep hygiene). Collaborative goal setting will be used to develop a bedtime routine and improve sleep quality, considering individual preferences and readiness for change. In session 4, participant’s individual beliefs about the cause and improvement of their back pain will be explored, with a non-judgmental and empathetic approach that aligns with the spirit of MI to understand their perspective. The focus is on eliciting their beliefs and exploring any ambivalence regarding the potential factors contributing to their back pain and its improvement. Finally, in Session 5, participants will be encouraged to reflect on the scenario where they provide advice to their future selves about dealing with back pain. This will focus on translating advice into a personalized action plan, setting clear goals, and emphasizing self-efficacy for managing and preventing future back pain episodes.

In the *Edu-only* intervention, standard essential resource materials are provided, in commonly used directive manner that will actively refrain from offering individualized support or incorporating MI-CBT elements. Specifically, research clinicians will actively listen, express interest, and provide undivided attention to participants without employing specific MI-CBT-based support or tailored pain education advice. Instead, participants will be directed to utilize the 'Navigating Pain' booklet in its standard form or receive answers directly quoted from its content, without customization or additional guidance. Furthermore, they will be advised to adhere to the exercise program. This approach distinguishes the *Edu-only* intervention by its emphasis on question–answer driven discussions and the deliberate absence of MI-CBT elements, ensuring a clear differentiation from the MI-CBT intervention group.

The two researchers undertaking data collection will undergo training on MI-CBT practises, involving the equivalent of a 2-day initial training by a Motivational Interviewing Network of Trainers (MINT) qualified trainer, and thereafter 2 sessions of individualised training. The trial will be conducted at one site, and adherence to protocol will be monitored by the larger research team.

### Intervention fidelity

Proficiency in the delivery of MI will be assessed using the Motivational Interviewing Treatment Integrity scale 4.2.1 (MITI 4.2.1) [42], and the Behaviour Change Counselling Index (BCCI) will be used to assess researcher competence in eliciting participants thoughts and cognitions, addressing the CBT strategies of the intervention [43]. Twenty random intervention sessions (10 from each group) will be audio-recorded for analysis of intervention fidelity at various time points during the trial, including confirmation that MI-CBT techniques were not used in the *Edu-only* group. A third-party assessor trained in the use of the MITI and BCCI, and not otherwise associated with the study, will review, and score the interactions.

Participants are permitted to seek any treatment needed during the study, and details of treatment for low back pain will be collected. The allocated intervention will be discontinued if the participant chooses to withdraw for any reason, can no longer physically complete the intervention, and/or meets the exclusion criteria during the trial. In accordance with the Health and Disability Ethics Committee (HDEC) standard, any risk to the participants will be mitigated, for example, for any scores > 10 on the HADS, a registered psychologist from the research team will contact the participant to provide further guidance and support. If the research team have any further concerns, this will be raised within 24 h of notification to the Director of the community clinic, and further support can be provided through the normal clinical operational protocols [44].

### Sample size estimation

The sample size for the main trial is 166 (*n* = 83 per intervention group) based on a 1.0 ± 2.0-point difference in numeric pain rating at 12-months between groups, which is suggested to be the minimum difference to detect a clinically meaningful change. Sample size estimation (1-ß = 0.80, α = 0.05) included group x time interactions over the 3 time points and allowed for a 15% drop-out [45].

### Ethics and management

A detailed description of the data to be collected at each time point (3-, 6- and 12-months) is presented in Table 1. All participants will be informed of, and provide consent for, the collection and use of their data for the purposes of this study, and for any mandatory secondary uses. Participants’ privacy and confidentiality will be respected through the protection of their data. The researchers will comply with legal and regulatory requirements regarding the privacy and confidentiality of participants’ data. Written consent forms, which will contain identifiable data, will be stored in a separate, secure location from the de-identified health information, accessible only to members of the research team. All paper-based documentation will be stored in a secure cabinet at the research site, and electronic documentation will be stored on a password-protected cloud-based server. All documents and data will be shredded or deleted 10 years after the study has been completed. The study has been approved by the Ministry of Health (New Zealand) Health and Disability Ethics Committee (HDEC reference: 2023 EXP 16691).

## Statistical methods

Data will be analysed by a statistician who is blinded to group membership. The primary analyses will be by intention-to-treat for any missing data using the last-point-carried-forward method. Multiple-mediation analysis with bias-corrected bootstrapping will be used to explore how changes in participant self-report explains relationships between group (Edu + MI-CBT vs. Edu) and 12-month outcomes for back pain intensity and disability, with healthcare seeking behaviours (i.e., patient reported number of treatments and medication use) examined as potential moderators of observed effects. Statistical significance will be set at *p* ≤ 0.05.

## Discussion

Low back pain is one of the most commonly reported reasons for pain in all age groups and remains the leading global cause of overall disability [46]. Globally, it is a major public health concern [2], therefore effective delivery of self-management strategies to prevent recurrence of low back pain requires urgent attention. For non-specific back pain, generally recommended treatments include education and advice to remain active, as well as exercise and cognitive behavioural therapy. In their recent Lancet series, Foster et al. [11] highlight that substantial discrepancies exist between evidence and current global practice. These authors also emphasize the importance of effectively implementing best known practice to improve outcomes and reduce cost. The current study will provide new information on the effectiveness of using an MI-CBT approach to facilitate back pain education, advice and exercise therapy to improve low back pain recurrence rates. The results of the study might be useful in restructuring health professional’s methods of management for chronic low back pain in New Zealand. There is a need for effective delivery of best practice recommendations that are context specific yet cost effective and scalable.

## Trial status

Recruitment is due to commence for this study in November 2023. The current protocol is version 3 (dated: 14/11/2023).

## Supplementary Information

Supplementary Material 1: Supplementary File 1. SPIRIT checklist for standardised reporting of clinical trial protocols.

Supplementary Material 2: Supplementary File 2. Participant information sheet and consent form.

Supplementary Material 3: Supplementary File 3: Back Pain Homework book resource.

## Data Availability

Data may be available on request to MK. Protocol will be published and publicly accessible. The protocol and results will be presented at international scientific conferences and in peer-reviewed publications.

## Abbreviations

BCCI: Behaviour Change Counselling Index
CBT: Cognitive behavioural therapy
FABQ: Fear-Avoidance Beliefs Questionnaire
GCP: Good Clinical Practice
HADS: Hospital Depression and Anxiety Score
HDEC: Health and Disability Ethics Committee
ODI: Oswestry Disability Index
PSEQ: Pain Self-Efficacy Questionnaire
MITI: Motivational Interviewing Treatment Integrity
MI: Motivational interviewing
MINT: Motivational Interviewing Network of Trainers
NZ: New Zealand

## Data availability

No datasets were generated or analysed during the current study.

## References

[1] Murray CJ, Lopez AD. Measuring the global burden of disease. N Engl J Med. 2013;369(5):448–57. 23902484 10.1056/NEJMra1201534

[2] Chen S, Chen M, Wu X, Lin S, Tao C, Cao H, Shao Z, Xiao G. Global, regional and national burden of low back pain 1990–2019: A systematic analysis of the Global Burden of Disease study 2019. J Orthop Transl. 2022;32:49–58. 34934626 10.1016/j.jot.2021.07.005 PMC8639804

[3] Ferreira ML, de Luca K, Haile LM, Steinmetz JD, Culbreth GT, Cross M, Kopec JA, Ferreira PH, Blyth FM, Buchbinder R. Global, regional, and national burden of low back pain, 1990–2020, its attributable risk factors, and projections to 2050: a systematic analysis of the Global Burden of Disease Study 2021. Lancet Rheumatology. 2023;5(6):e316–29. 37273833 10.1016/S2665-9913(23)00098-X PMC10234592

[4] Deyo RA, Weinstein JN. Low back pain. N Engl J Med. 2001;344(5):363–70. 11172169 10.1056/NEJM200102013440508

[5] Carey TS, Garrett JM, Jackman A, Hadler N, Project TNCBP. Recurrence and care seeking after acute back pain: results of a long-term follow-up study. Med Care. 1999;37(2):157–64. 10.1097/00005650-199902000-00006 10024120

[6] Croft PR, Macfarlane GJ, Papageorgiou AC, Thomas E, Silman AJ. Outcome of low back pain in general practice: a prospective study. BMJ. 1998;316(7141):1356. 9563990 10.1136/bmj.316.7141.1356 PMC28536

[7] Da Silva T, Mills K, Brown BT, Herbert RD, Maher CG, Hancock MJ. Risk of recurrence of low back pain: a systematic review. J Orthop Sports Phys Ther. 2017;47(5):305–13. 28355981 10.2519/jospt.2017.7415

[8] Traeger AC, Lee H, Hübscher M, Skinner IW, Moseley GL, Nicholas MK, Henschke N, Refshauge KM, Blyth FM, Main CJ. Effect of intensive patient education vs placebo patient education on outcomes in patients with acute low back pain: a randomized clinical trial. JAMA Neurol. 2019;76(2):161–9. 30398542 10.1001/jamaneurol.2018.3376 PMC6440280

[9] Ferreira GE. Lin C-WC, Stevens ML, Hancock MJ, Latimer J, Kelly P, Wisbey-Roth T, Maher CG: Exercise is medicine, but perhaps not for preventing low back pain: A randomized trial of exercise and education to prevent low back pain recurrence. J Orthop Sports Phys Ther. 2021;51(4):188–95. 33789433 10.2519/jospt.2021.10187

[10] Traeger AC, Henschke N, Hübscher M, Williams CM, Kamper SJ, Maher CG, Moseley GL, McAuley JH. Estimating the risk of chronic pain: development and validation of a prognostic model (PICKUP) for patients with acute low back pain. PLoS Med. 2016;13(5): e1002019. 27187782 10.1371/journal.pmed.1002019 PMC4871494

[11] Foster NE, Anema JR, Cherkin D, Chou R, Cohen SP, Gross DP, Ferreira PH, Fritz JM, Koes BW, Peul W. Prevention and treatment of low back pain: evidence, challenges, and promising directions. Lancet. 2018;391(10137):2368–83. 10.1016/S0140-6736(18)30489-6 29573872

[12] Bernstein IA, Malik Q, Carville S, Ward S. Low back pain and sciatica: summary of NICE guidance. BMJ. 2017;356: i6748. 10.1136/bmj.i6748. 28062522 10.1136/bmj.i6748

[13] Oliveira CB, Maher CG, Pinto RZ, Traeger AC. Lin C-WC, Chenot J-F, van Tulder M, Koes BW: Clinical practice guidelines for the management of non-specific low back pain in primary care: an updated overview. Eur Spine J. 2018;27:2791–803. 29971708 10.1007/s00586-018-5673-2

[14] Koes BW, van Tulder M, Lin CW, Macedo LG, McAuley J, Maher C. An updated overview of clinical guidelines for the management of non-specific low back pain in primary care. Eur Spine J. 2010;19(12):2075–94. 20602122 10.1007/s00586-010-1502-y PMC2997201

[15] van Koppen B, Zandwijk P, de Vries J, van Mameren H, de Bie R. Adherence to home-based exercises and/or activity advice in low back pain patients: a systematic review. Eur J Physiother. 2022;24(4):227–42. 10.1080/21679169.2020.1846783

[16] Jordan JL, Holden MA, Mason EE, Foster NE. Interventions to improve adherence to exercise for chronic musculoskeletal pain in adults. Cochrane Database Syst Rev. 2010(1):CD005956. 10.1002/14651858.CD005956.pub2. 10.1002/14651858.CD005956.pub2 PMC6769154 20091582

[17] Steffens D, Maher CG, Pereira LS, Stevens ML, Oliveira VC, Chapple M, Teixeira-Salmela LF, Hancock MJ. Prevention of Low Back Pain: A Systematic Review and Meta-analysis. JAMA Intern Med. 2016;176(2):199–208. 26752509 10.1001/jamainternmed.2015.7431

[18] O’Halloran P, Blackstock F, Shields N, Holland A, Iles R, Kingsley M, Bernhardt J, Lannin N, Morris ME, Taylor NF. Motivational interviewing to increase physical activity in people with chronic health conditions: a systematic review and meta-analysis. Clin Rehabil. 2014;28(12):1159–71. 24942478 10.1177/0269215514536210

[19] Miller WR, Rollnick S. Motivational Interviewing: Helping People Change, 3rd ed. New York: Guilford Press; 2012.

[20] Frey AJ, Lee J, Small JW, Sibley M, Owens JS, Skidmore B, Johnson L, Bradshaw CP, Moyers TB. Mechanisms of motivational interviewing: A conceptual framework to guide practice and research. Prev Sci. 2021;22:689–700. 32666269 10.1007/s11121-020-01139-x

[21] Armstrong MJ, Mottershead TA, Ronksley PE, Sigal RJ, Campbell TS, Hemmelgarn BR. Motivational interviewing to improve weight loss in overweight and/or obese patients: a systematic review and meta-analysis of randomized controlled trials. Obes Rev. 2011;12(9):709–23. 21692966 10.1111/j.1467-789X.2011.00892.x

[22] Hettema J, Steele J, Miller WR. Motivational interviewing. Annual Review. Clinical Psychology. 2005;1:91–111. 10.1146/annurev.clinpsy.1.102803.143833 17716083

[23] Smedslund G, Berg RC, Hammerstrom KT, Steiro A, Leiknes KA, Dahl HM, Karlsen K. Motivational interviewing for substance abuse. Campbell Syst Rev. 2011;7(1):1–126. 10.4073/csr.2011.6. 10.4073/csr.2011.6 PMC8939890 21563163

[24] Barrett S, Begg S, O’Halloran P, Kingsley M. Integrated motivational interviewing and cognitive behaviour therapy can increase physical activity and improve health of adult ambulatory care patients in a regional hospital: the Healthy4U randomised controlled trial. BMC Public Health. 2018;18:1166. 10.1186/s12889-018-6064-7. 30305078 10.1186/s12889-018-6064-7 PMC6180400

[25] Thompson D, Chair S, Chan S, Astin F, Davidson P, Ski C. Motivational interviewing: A useful approach to improving cardiovascular health? J Clin Nurs. 2011;20:1236–44. 21492271 10.1111/j.1365-2702.2010.03558.x

[26] Lee WWM, Choi KC, Yum RWY, Yu DSF, Chair SY. Effectiveness of motivational interviewing on lifestyle modification and health outcomes of clients at risk or diagnosed with cardiovascular diseases: a systematic review. Int J Nurs Stud. 2016;53:331–41. 10.1016/j.ijnurstu.2015.09.010 26493130

[27] Iles R, Taylor NF, Davidson M, O’Halloran P. Telephone coaching can increase activity levels for people with non-chronic low back pain: a randomised trial. J Physiother. 2011;57(4):231–8. 22093121 10.1016/S1836-9553(11)70053-4

[28] Leonhardt C, Keller S, Chenot J-F, Luckmann J, Basler H-D, Wegscheider K, Baum E, Donner-Banzhoff N, Pfingsten M, Hildebrandt J. TTM-based motivational counselling does not increase physical activity of low back pain patients in a primary care setting—a cluster-randomized controlled trial. Patient Educ Couns. 2008;70(1):50–60. 18023130 10.1016/j.pec.2007.09.018

[29] Basler H-D, Bertalanffy H, Quint S, Wilke A, Wolf U. TTM-based counselling in physiotherapy does not contribute to an increase of adherence to activity recommendations in older adults with chronic low back pain–a randomised controlled trial. Eur J Pain. 2007;11(1):31–7. 16448828 10.1016/j.ejpain.2005.12.009

[30] Vong SK, Cheing GL, Chan F, So EM, Chan CC. Motivational enhancement therapy in addition to physical therapy improves motivational factors and treatment outcomes in people with low back pain: a randomized controlled trial. Arch Phys Med Rehabil. 2011;92(2):176–83. 21272712 10.1016/j.apmr.2010.10.016

[31] Gatchel RJ, Rollings KH. Evidence-informed management of chronic low back pain with cognitive behavioral therapy. Spine J. 2008;8(1):40–4. 18164452 10.1016/j.spinee.2007.10.007 PMC3237294

[32] Richmond H, Hall AM, Copsey B, Hansen Z, Williamson E, Hoxey-Thomas N, Cooper Z, Lamb SE. The effectiveness of cognitive behavioural treatment for non-specific low back pain: a systematic review and meta-analysis. PLoS ONE. 2015;10(8): e0134192. 26244668 10.1371/journal.pone.0134192 PMC4526658

[33] Darnall BD, Roy A, Chen AL, Ziadni MS, Keane RT, You DS, Slater K, Poupore-King H, Mackey I, Kao M-C. Comparison of a single-session pain management skills intervention with a single-session health education intervention and 8 sessions of cognitive behavioral therapy in adults with chronic low back pain: a randomized clinical trial. JAMA Netw Open. 2021;4(8):e2113401–e2113401. 34398206 10.1001/jamanetworkopen.2021.13401 PMC8369357

[34] Butcher NJ, Monsour A, Mew EJ, Chan A-W, Moher D, Mayo-Wilson E, Terwee CB, Chee-A-Tow A, Baba A, Gavin F. Guidelines for reporting outcomes in trial protocols: the SPIRIT-outcomes 2022 extension. JAMA. 2022;328(23):2345–56. 36512367 10.1001/jama.2022.21243

[35] Suzuki H, Aono S, Inoue S, Imajo Y, Nishida N, Funaba M, Harada H, Mori A, Matsumoto M, Higuchi F. Clinically significant changes in pain along the Pain Intensity Numerical Rating Scale in patients with chronic low back pain. PLoS ONE. 2020;15(3): e0229228. 32126108 10.1371/journal.pone.0229228 PMC7053735

[36] Franchignoni F, Giordano A, Ferriero G, Monticone M. Measurement precision of the Pain Catastrophizing Scale and its short forms in chronic low back pain. Sci Rep. 2022;12(1):12042. 35835830 10.1038/s41598-022-15522-x PMC9283330

[37] Waddell G, Newton M, Henderson I, Somerville D, Main CJ. A Fear-Avoidance Beliefs Questionnaire (FABQ) and the role of fear-avoidance beliefs in chronic low back pain and disability. Pain. 1993;52(2):157–68. 8455963 10.1016/0304-3959(93)90127-B

[38] Nicholas MK. The pain self-efficacy questionnaire: taking pain into account. Eur J Pain. 2007;11(2):153–63. 16446108 10.1016/j.ejpain.2005.12.008

[39] Zigmond AS, Snaith RP. The hospital anxiety and depression scale. Acta Psychiatr Scand. 1983;67(6):361–70. 6880820 10.1111/j.1600-0447.1983.tb09716.x

[40] Vianin M. Psychometric properties and clinical usefulness of the Oswestry Disability Index. J Chiropr Med. 2008;7(4):161–3. 19646379 10.1016/j.jcm.2008.07.001 PMC2697602

[41] New Zealand Pain Society. Navigating Pain: Finding the right path for your pain management journey. https://www.nzps.org.nz/assets/2ee400518f/2204-Pain-Management-Resource-A5-FINAL-online.pdf . Accessed 19 Jul 2023.

[42] Moyers TB, Rowell LN, Manuel JK, Ernst D, Houck JM. The motivational interviewing treatment integrity code (MITI 4): rationale, preliminary reliability and validity. J Subst Abuse Treat. 2016;65:36–42. 26874558 10.1016/j.jsat.2016.01.001 PMC5539964

[43] Vallis M. Behaviour change counselling—How do I know if I am doing it well? The development of the Behaviour Change Counselling Scale (BCCS). Can J Diabetes. 2013;37(1):18–26. 24070744 10.1016/j.jcjd.2013.01.005

[44] International Ethical Guidelines for Health-related Research Involving Humans, Fourth Edition. Geneva. Council for International Organizations of Medical Sciences (CIOMS); 2016. https://cioms.ch/wp-content/uploads/2017/01/WEB-CIOMS-EthicalGuidelines.pdf. Accessed 16 Jun 2023. 40523065

[45] Faul F, Erdfelder E, Lang A-G, Buchner A. G*Power 3: A flexible statistical power analysis program for the social, behavioral, and biomedical sciences. Behav Res Methods. 2007;39:175–91. 17695343 10.3758/BF03193146

[46] Buchbinder R, Underwood M, Hartvigsen J, Maher CG. The Lancet Series call to action to reduce low value care for low back pain: an update. Pain. 2020;161(1):S57. 33090740 10.1097/j.pain.0000000000001869 PMC7434211

