# Supplementary material for: Breaking the cycle of reoccurring low back pain with integrated motivational interviewing and cognitive behavioural therapy to facilitate education and exercise advice: a superiority randomised controlled trial study protocol
Source: BMC Public Health. 2024 Sep 5;24:2415. doi: 10.1186/s12889-024-19930-8 (PMC11375947; doi:10.1186/s12889-024-19930-8)
Supplement: Supplementary file 2 — Supplementary Material 2: Supplementary File 2. Participant information sheet and consent form. [file 12889_2024_19930_MOESM2_ESM.doc]

| Participant Information Sheet **Preventing the recurrence of low back pain** | | |  | |
| --- | --- | --- | --- | --- |
| Sponsor: NZ Lotteries Health Research Fund: The University of Auckland: New Zealand College of Chiropractic |  | | | |
| Lead Researcher:XXXX, University of Auckland (xxxx)  Study Site: Chiropractic Clinical Center, New Zealand College of Chiropractic  Contact phone number: xxxxx  Ethics committee ref: xxxxx |  |  | |  |
|  |  |  | |  |

You are invited to take part in a study looking at how we can prevent someone having more back pain in the next 12-months. Whether or not you take part is your choice. If you don’t want to take part, you don’t have to give a reason, and it won’t affect the care you receive. If you do want to take part now, but change your mind later, you can pull out of the study at any time.

This Participant Information Sheet will help you decide if you’d like to take part. It sets out why we are doing the study, what your participation would involve, what the benefits and risks to you might be, and what would happen after the study ends. We will go through this information with you and answer any questions you may have. You do not have to decide today whether or not you will participate in this study. Before you decide you may want to talk about the study with other people, such as family, whānau, friends, or healthcare providers. Feel free to do this.

If you agree to take part in this study, you will be asked to sign the Consent Form on the last page of this document. You will be given a copy of both the Participant Information Sheet and the Consent Form to keep.

This document is 9-pages long, including the Consent Form. Please make sure you have read and understood all the pages.

| **Voluntary Participation and Withdrawal From This Study** |
| --- |

Your participation in this research is voluntary. If you don’t want to take part, you don’t have to give a reason. Whether or not you choose to participate will neither advantage nor disadvantage you. If you do want to take part now, but change your mind later, you can pull out of the study at any time.

## What is the purpose of the study?

This study will examine how education about pain and how it might impact your life as well as physical activity advice might help people who have recovered from an episode of low back pain. We hope to provide evidence that might be used to help how chiropractors and physiotherapists talk with back pain patients after they have finished treatment, to try and stop the back pain returning.

| **How is the study designed and what does my participation involve?** |
| --- |

The first part of our study will provide the patient education and physical activity advice to 15 people, then to talk with these participants about their thoughts on our study and how it might be improved. This part of our study will include 15 people, who will attend two 30-minute sessions (one-week apart) with a member of the research team. Each session will involve the researcher talking with you about what you have been doing for your back pain, giving you some education and advice about managing any pain in the future, and providing physical activity advice to help prevent back pain in the future. Every participant in this study will also receive the *‘Navigate Pain’* booklet from the NZ Pain Society, which contains essential information to help your recovery. After the first session we will also give you a ‘homework’ book to see what your thoughts are on the types of tasks that people often say hurt their back (e.g., what if you do 8-hours gardening next weekend?). After both sessions we will interview the people who took part (1:1 with a research team member, 30 mins) to get their thoughts on the intervention, and what we could do to improve it for the main trial. This interview will be recorded.

For the main trial we will recruit 170 people who have recovered from back pain after receiving treatment at the New Zealand Chiropractic Centre. We will provide you the ‘updated’ education and physical activity advice over the two 30-minute sessions (including giving you the homework book to fill in between the two sessions), and conduct two more 30-minute sessions in two- and four-weeks via your preferred method of catching-up (e.g., in-person, Zoom, phone). Our goal is to help provide you education about back pain and physical activity to help your recovery and prevent any future back pain episodes.

For people in the main study, we will look to contact you at 3-, 6-, and 12-months to ask how you’re feeling, and to get you to fill in some forms about any pain you might have, and how you’re handling different parts of life. These forms can be mailed out to you (hard-copy), or sent over email to be completed and returned via an attached electronic document. These forms ask about how you feel about back pain (even if you don’t have it), how you’re feeling in life, and what types of things you’ve been doing in your life for physical activity (and if you’ve had to have any treatment because your back pain came back).

| **Who can take part in the study?** |
| --- |

You are invited to take part in this study if you have recovered from an episode of low back pain after treatment at the NZ Chiropractic Centre, and are over the age of 18. This must have been the first back pain you can remember having in the last year that you have had to do something about (e.g., take pain medication, seek treatment). If you’ve had a sore back after sitting for too long, sleeping the wrong way, and it got better soon after without getting treatment you can still take part in this study. Unfortunately the back pain you have just received treatment for cannot be from something specific (e.g., a disc herniation, structural damage), and you cannot have any other diseases or disabilities that might have contributed to your back pain or would require you to see someone else for your health (e.g., if you have diagnosed heart disease you can’t take part in this study). Please contact a research team member to discuss your potential participation in your study. A simple way to check your recovery is if your back pain has been less than 3 out of 10 for the last 7 days in a row. If it has, then you are eligible to be part of this study.

## What am I asked to complete?

If you are in the main study, here is a summary of the self-report assessment items we will ask you to complete at the start of the study, and after 3-, 6-, and 12-months. On the consent form at the end of this document please indicate how you’d prefer we provide these to you at the 3-, 6-, and 12-month time points. If we notice a score that is higher than what we would expect for this population on the Depression and Anxiety score, we will reach out to you and encourage you to go and talk with your General Practitioner or other another Allied Health Professional.

| Oswestry Disability Index  Pain Catastrophizing Questionnaire | Numeric Rating Pain Scale  Hospital Anxiety and Depression Score | Fear-Avoidance Beliefs Questionnaire  Pain Self-Efficacy Questionnaire |
| --- | --- | --- |

Another part of our study is we want to make sure our clinicians are delivering the intervention in the way it was designed. To this end, we would like to record (at random) the first session of 20 participants in this study. On the consent form we will ask if you consent to the audio of your first session being recorded as part of this study.

## What are the possible risks of this study?

There are no foreseeable risks to you for participating in this study. You are allowed to engage with the education and physical activity we will provide at your own discretion (i.e., you don’t actually have to do any of it – we’d prefer you did, but it is your choice). You are also allowed to engage with any other type of treatment you might decide is necessary for your back pain, but the risks associated with that are outside the scope of this study.

| **What are the possible benefits of this study?** |
| --- |

We expect to reduce both the recurrence rates of back pain in the people who take part in this study, as well as the level of pain and general well-being of participants. Based on published data we expect any pain or disability you are currently experiencing to be reduced by another 30% by 3-months, with this maintained up to 12-months. Data suggests that around 30% of participants in this study will report some type of chronic symptoms at 12-months, but this is much better than the normal recurrence of pain symptoms which is typically around 50%.

| **What are the alternatives to taking part?** |
| --- |

You are free to pursue any other type of ongoing treatment for your back pain. Even if you do agree to take part in this study, we will not stop you from pursuing any other type of treatment you would like for any back pain symptoms you have (as long as you let us know what you’re doing when we catch-up with you during the next 12-months).

## Will any costs be reimbursed?

There are no costs for you being associated with this study. The first two sessions we want to have with you will be at the NZ Chiropractic Centre where you have already been receiving treatment, so there are no parking costs for you.

## What if something goes wrong?

If you are injured during the course of this study, you would be eligible to apply for compensation from ACC just as you would be if you were injured in an accident at work or at home. This does not mean that your claim will automatically be accepted. You will have to lodge a claim with ACC, which may take some time to assess. If your claim is accepted, you will receive funding to assist in your recovery.

If you have private health or life insurance, you may wish to check with your insurer that taking part in this study won’t affect your cover.

## What will happen to my information?

During this study the research team will record information about you and your study participation. This includes the results of the study assessments, anything you choose to fill in the homework book, the interview about your experiences (if you are in the first part of the study with 15 people), and recordings of your first session (you can consent for this separately, and we are only using 20 random recordings to see how well the clinicians are talking with you). You cannot take part in this study if you do not consent to the collection of this information (you do not have to consent to having your treatment session recorded).

Identifiable Information

Identifiable information is any data that could identify you (e.g. your name, date of birth, or address). Only the research team will have access to identifiable information collected in this study.

De-identified (Coded) Information

To make sure your personal information is kept confidential, information that identifies you will not be included in any report generated by the research team. Instead, you will be identified by a code. Dr. Paul Marshall will keep a list linking your code with your name and the results from the self-report forms you complete during this study, so that you can be identified by your coded data if needed. It must be noted that at the completion of data collection, this dataset will be completely deidentified prior to any subsequent analysis so your specific name and details cannot be linked with any other data collected in this study. This means if you want to withdraw from the study (which you can at any time without giving a reason), you must do this before completion of the 12-month follow-ups.

The results of the study may be published or presented, but not in a form that would reasonably be expected to identify you.

Your information will not be used for future research.

Security and Storage of Your Information.

Your identifiable information and any hard-copies of completed self-report forms will be stored in a locked cabinet at Dr. Imran Khan Niazi’s office in New Zealand College of Chiropractic during the study. After the study it is transferred to a locked cabinet at the Department of Exercise Sciences (Research Storage Space, Building 907, University of Auckland) and stored for 10 years, then destroyed. Any self-report forms completed via email, and any electronic audio recordings from your involvement in the study will be securely stored by Dr. Paul Marshall on the password protected University of Auckland server for 10-years, then deleted. Your coded information will be entered into an electronic datasheet that will be kept securely stored by Dr. Paul Marshall on the University of Auckland’s password protected server. Coded study information will be kept by Dr. Marshall for 10 years, then deleted. All storage will comply with local and/or international data security guidelines.

Risks.

Although efforts will be made to protect your privacy, absolute confidentiality of your information cannot be guaranteed. Even with coded and anonymised information, there is no guarantee that you cannot be identified. The risk of people accessing and misusing your information (e.g., making it harder for you to get or keep a job or health insurance) is currently very small, but may increase in the future as people find new ways of tracing information.

Rights to Access Your Information.

You have the right to request access to your information held by the research team. You also have the right to request that any information you disagree with (e.g., transcription of a recorded interview) is corrected. However, most of the data will be de-identified and grouped with other participants’ data. If you have any questions about the collection and use of information about you, you should ask the lead researcher.

Rights to Withdraw Your Information.

You may withdraw your consent for the collection and use of your information at any time, by informing any of the research team.

If you withdraw your consent, your study participation will end, and the study team will stop collecting information from you. However, information collected up until your withdrawal from the study will continue to be used and included in the study.

Māori Data Sovereignty

Māori data sovereignty is about protecting information or knowledge that is about (or comes from) Māori people. We recognise the taonga of the data collected for this study. The information from the participants will be treated with respect and will be stored in Aotearoa, New Zealand.`

**What happens after the study or if I change my mind?**

**What if I change my mind?**

You can withdraw from the study at any time by notifying any of the research team. If you choose to withdraw from the study, then you will be offered the choice between having any data that is identifiable as belonging to you removed or allowing it to continue to be used. However, once the findings have been produced, the removal of your data may not be possible.

**What happens after the study?**

Your record will be securely stored by Dr. Paul Marshall at the Department of Exercise Sciences, University of Auckland. After ten years, all original data collection sheets will be shredded, and the electronic data including audio recordings will be deleted.

You will receive a summary report at the end of this study describing the overall results and what we found. There will be no information in this report that could be used to identify you at all. If you would like to discuss the results of this study or anything about your experiences in this study, please contact Dr. Paul Marshall so this can be arranged.

| **Who is funding the study?** |
| --- |

This study is being funded by the New Zealand Lotteries Health Project Grant (LHR 2023-215974). The affiliations of our research team members include the University of Auckland, NZ Chiropractic College, Western Sydney University, and LaTrobe University (Australia).

| **Who Has Approved the study?** |
| --- |

This study has been approved by an independent group of people called a Health and Disability Ethics Committee (HDEC), who check that studies meet established ethical standards. The [insert Committee name] has approved this study.

## Who do I contact for more information or if I have concerns?

If you have any questions, concerns or complaints about the study at any stage, you can contact any of the following research team members:

*xxxxxx*

*xxxxxx*

If you want to talk to someone who isn’t involved with the study, you can contact an independent health and disability advocate on:

Phone: 0800 555 050
Fax: 0800 2 SUPPORT (0800 2787 7678)
Email: [advocacy@advocacy.org.nz](mailto:advocacy@advocacy.org.nz)

Website: https://www.advocacy.org.nz/

For Māori cultural support please talk to your whānau in the first instance. You may also contact the administrator for He Kamaka Waiora (Māori Health Team) by telephoning 09 486 8324 ext 2324

You can also contact the health and disability ethics committee (HDEC) that approved this study on:
Email: [hdecs@health.govt.nz](mailto:hdecs@health.govt.nz)

Phone: 0800 400 569 (Ministry of Health general enquiries)

**Consent Form**

**Preventing the recurrence of low back pain**

**Please tick to indicate you consent to the following**

| I have read the Participant Information Sheet, or have had it read to me in a language I understand, and I fully comprehend what it says. |  |  |
| --- | --- | --- |
| I have been given sufficient time to consider whether or not to participate in this study. |  |  |
| I have had the opportunity to use a legal representative, whanau/ family support or a friend to help me ask questions and understand the study. |  |  |
| I am satisfied with the answers I have been given regarding the study and I have a copy of this consent form and information sheet. |  |  |
| I understand that taking part in this study is voluntary (my choice) and that I may withdraw from the study at any time without this affecting my medical care. |  |  |
| I understand that if I am part of the first 15 people recruited for this study, I am in the ‘feasibility’ phase and I consent to being interviewed about my experiences after receiving the treatment sessions. | Yes 🞏 | No 🞏 |
| I consent to the research staff collecting and processing my information, including information about my health. |  |  |
| If I decide to withdraw from the study, I agree that the information collected about me up to the point when I withdraw may continue to be processed. | Yes 🞏 | No 🞏 |
| I agree to an approved auditor appointed by the New Zealand Health and Disability Ethics Committees, or any relevant regulatory authority or their approved representative reviewing my relevant medical records for the sole purpose of checking the accuracy of the information recorded for the study. |  |  |
| I understand that my participation in this study is confidential and that no material, which could identify me personally, will be used in any reports on this study. |  |  |
| I understand that after the 12-month assessment my data will be deidentified and I will no longer be able to withdraw information collected about me. |  |  |
| I consent to the researchers recording my sessions with the clinicians to see if the intervention is being delivered as designed (this will only happen for 20 people at random). | Yes 🞏 | No 🞏 |
| I understand the compensation provisions in case of injury during the study. |  |  |
| I know who to contact if I have any questions about the study in general. |  |  |
| I understand my responsibilities as a study participant. These are my preferred contact details and method of contact for the follow-up assessments at 3-, 6-, and 12-months  Ph:  Email:  How I would like the follow-up forms provided to me  Email (as above)  Hard-Copy Address: |  |  |
| I wish to receive a summary of the results from the study. | Yes 🞏 | No 🞏 |

How would you like to receive a summary of the results?

Email (as above) / Hard-Copy (as above)

**Declaration by participant:**

I hereby consent to take part in this study.

| Participant’s name: | |
| --- | --- |
| Signature: | Date: |

**Declaration by member of research team:**

I have given a verbal explanation of the research project to the participant, and have answered the participant’s questions about it.

I believe that the participant understands the study and has given informed consent to participate.

| Researcher’s name: | |
| --- | --- |
| Signature: | Date: |
