# Supplementary material for: Breaking the cycle of reoccurring low back pain with integrated motivational interviewing and cognitive behavioural therapy to facilitate education and exercise advice: a superiority randomised controlled trial study protocol
Source: BMC Public Health. 2024 Sep 5;24:2415. doi: 10.1186/s12889-024-19930-8 (PMC11375947; doi:10.1186/s12889-024-19930-8)
Supplement: Supplementary file 3 — Supplementary Material 3: Supplementary File 3: Back Pain Homework book resource. [file 12889_2024_19930_MOESM3_ESM.docx]

**Back Pain Homework Book**

(let us know if you would like to take the hard-copy home, or have a version emailed to you 😊 )

**What is this book?**

This homework book involves 5 ‘scenarios’ that are about you and your back pain. This is a good way for you to explore your thoughts about pain, and to help prepare you for managing what might happen in the future. You can respond to these however you like. If you would like to use the ***Navigate Pain*** book before or after looking at these scenarios, please feel free to do so. You can talk with anyone in your whānau about any of these scenarios and share anything you are thinking with them. At your session next week with our research team member, we would like to have a look at your ‘homework’, **but we will NOT be keeping this book**. No-one else will get to see what you have completed in this homework book. Please keep this with you in future and look back on these responses whenever you feel like it.

**Scenario 1: Understanding Pain**

Bob is used to hanging out the washing in bare feet. The washing line is on a concrete base, and Bob often stands on small rocks and feels a short dull ache which is annoying. One day he stands on a rock and reaches down to knock it away, but sees he’s actually been stung by a bee! He quickly flicks away the stinger like he was taught as a kid. A few weeks later Bob is out hanging out the washing, stands on something and he yells out with intense pain, thinking he’s been stung by a bee again. After sitting down on the ground he looks at his foot, and its just a pebble, not a bee sting. The pain is quickly gone.

What does this scenario mean to you about what pain can sometimes be from?

|  |
| --- |

**Scenario 2: Sleepy Time**

Its pretty common in life when things get busy that we might have a late-night for any number of reasons (e.g., work, whānau, Netflix) and end up falling into bed without much thought for a bed-time routine. Sometimes you might wake up in the morning with bit of a stiff or sore back, and feel a ‘bit slow’ getting moving for the day.

If this happens to you in future (waking up a bit stiff and sore), would you look to do anything about it for the next nights’ sleep?

**Scenario 3: Back Pain and Exercise**

There are lots of different exercise programs out there for people who have had low back pain, or have low back pain, with all kinds of different messages. Next week we will give you an exercise program we know works for people with back pain because we’ve tested it before in clinical trials. When you are out in the world though, some people will tell you your back pain is because your ‘core muscles are weak’; others might say your ‘muscles aren’t firing’. We know that physical activity is important for people with low back pain for all kinds of reasons. **What we’re interested in are your thoughts on whether physical activity or certain types of exercise are important for you to stop you having any more back pain in the future?**

|  |
| --- |

**Scenario 4: Back Pain and You**

Every year it is estimated that between 60-80% of all people will have some form of back pain. This is often not too bad and goes away. For you, you had back pain that required you to seek treatment from a chiropractor, and you have seen improvement in symptoms 😊 (otherwise you wouldn’t be in our study). Our research team has spoken to lots of people with back pain over the years, and have worked with lots of different clinicians and research teams about understanding what might cause back pain. What we’re interested in here is what do you think caused your back pain, and why have you had improvement in your symptoms already?

|  |
| --- |

**Scenario 5: Giving Advice to Future You 😊**

Despite the best efforts of your chiropractor who treated you and the support we’re trying to give you, we expect nearly half of people who have recovered from an episode of back pain will have another episode of back pain in the next year ☹ Take this scenario as an opportunity to tell future you what to do if you get back pain again. What treatment or life advice would you like to give future you? Is there a key message you’d like to pass along about treatment, or about the back pain, that you could record now and look at in future if you have another episode of back pain?

|  |
| --- |
